# The different role of intratumoral and peritumoral lymphangiogenesis in gastric cancer progression and prognosis

**DOI:** 10.1186/s12885-015-1501-9

**Published:** 2015-07-04

**Authors:** Kyung Ho Pak, Ara Jo, Hye Ji Choi, Younghee Choi, Hyunki Kim, Jae-Ho Cheong

**Affiliations:** 1 Department of Surgery, Hallym University Medical Center, Hwasung, South Korea; 2 Department of Pathology, Hallym University Medical Center, Hwasung, South Korea; 3 Department of Pathology, Yonsei University College of Medicine, 50-1 Yonsei-ro, Seodaemun-gu, 120-752 Seoul, South Korea; 4 Depatment of Surgery, Yonsei University College of Medicine, 50-1 Yonsei-ro, Seodaemun-gu, 120-752 Seoul, South Korea; 5 Department of Biochemistry & Molecular Biology, Yonsei University College of Medicine, 50-1 Yonsei-ro, Seodaemun-gu, 120-752 Seoul, South Korea; 6 Brain Korea 21 PLUS Project for Medical Science, Yonsei University College of Medicine, Seoul, South Korea

**Keywords:** Lymphangiogenesis, Lymphovascular density (LVD), Gastric cancer

## Abstract

**Background:**

Tumor-induced lymphangiogenesis plays a crucial role in metastasis and tumor progression. However, the significance of intratumoral lymphovascular density (I-LVD) and peritumoral lymphovascular density (P-LVD) has been controversial in gastric cancer. The purpose of this study was to investigate the differences of clinicopathologic characteristics with respect to I-LVD and P-LVD in gastric cancer.

**Methods:**

Samples of I-LVD and P-LVD from 66 patients who had undergone radical gastrectomy for gastric cancer were assessed after staining with D2-40, an immunostaining marker for lymphatic endothelium. The mean number of lymphatic vessels in three hotspots was calculated in intratumoral and peritumoral areas.

**Results:**

The peritumoral lymphatics were enlarged with dilated lumens compared to the intratumoral lymphatics. I-LVD was positively correlated with diffuse gastric cancer subtype, tumor stage, lymphovascular invasion, tumor node metastasis stage, and overall survival (*P* <0.05). P-LVD was associated with lymphovascular invasion, node stage, and disease-free survival (*P* <0.05).

**Conclusions:**

We conclude that P-LVD had an important role in lymph node metastasis, while I-LVD was more associated with depth of tumor invasion. However, both LVDs contributed to gastric cancer progression and prognosis.

## Background

In 2012, gastric cancer was responsible for 723,000 deaths and was ranked as the world’s third leading cause of cancer mortality [1]. Gastric cancer was also the second most common malignancy in Korea [2]. Regional lymph nodes are the most common site of tumor spread and lymph node metastasis is a major prognostic factor in gastric carcinomas. Thus, understanding the mechanism of lymphatic metastasis represents a crucial step that could result in a new therapeutic target in the treatment of gastric cancer.

Recent studies have suggested that lymphangiogenesis, the formation of new lymphatic vessels induced by tumors, is directly correlated with the extent of metastasis of solid tumors in lymph nodes [3–8]. Lymphatic vessel density (LVD) is a quantitative measure of tumor lymphangiogenesis and is measured by directly counting lymphatic vessels. It has been reported that high LVD in gastric cancer correlates with regional lymph node metastasis and poor prognosis [9–11]. However, in these studies, the effect of lymphangiogenesis associated with intratumoral lymphatics or peritoumoral lymphatics was not evaluated.

There is considerable debate about the roles of intratumoral versus peritumoral lymphatics in the pathology of the primary tumor [3, 5, 8, 12–16]. It is well established that peritumoral lymphatics are predominantly responsible for providing access to cancer cells during metastasis [16]. Peritumoral-LVD (P-LVD) was associated with lymph node metastases of tumors, for example, of the breast, prostate, and uterine cervix [3, 5, 8]. In contrast, intratumoral-LVD (I-LVD) was predictive of lymphatic metastasis in cancers of other organs, including papillary carcinoma of the thyroid and squamous cell carcinoma of the head, neck, esophagus, and oral cavity [12–15]. Nonetheless, the role of different lymphatics in gastric cancer is unclear and remains controversial. Some researchers have concluded that P-LVD is more important than I-LVD [17–19], while others present conflicting results [20–23].

Here, we investigated the clinical significance of I-LVD and P-LVD in gastric cancer to evaluate their role as risk factors for lymph node metastasis, disease recurrence, and overall survival.

## Methods

### Patients and specimens

Three hundred and sixty-five patients with gastric cancer underwent curative radical gastrectomy at Severance Hospital of Yonsei University, Seoul, Korea during the period of June 2005 to December 2005. This is the longest follow-up period that we are aware of in which complete data, including lymphovascular invasion (LVI) status, was collected. For preliminary data to test a correlation between the location of lymphatics and clinicopathologic parameters, we selected 66 cases from the 365 cases with their Tumor, Node, Metastasis (TNM) stages according to Lauren’s classification. None of the patients these specimens came from had received pre-operative chemotherapy or radiotherapy. The study population consisted of 47 men (71 %) and 19 women (19 %). The patients’ ages ranged from 23 to 81 years and the average age at diagnosis was 59 years. There were 18 cases of early gastric cancer and 48 cases of advanced gastric cancer. The histological stage was based on classification system cited in the American Joint Committee on Cancer’s Staging Manual, 7^th^ edition. Other clinical features assessed and are summarized in Table 1. Patients were followed up clinically for at least five years after surgery, except in mortality cases. The follow-up time ranged from 3 to 69 months, with an average follow-up time of 55 months. The current study was approved by the Institutional Review Board of Severance Hospital, Yonsei University (4-2012-0427).

**Table 1:** Correlation of LVD with clinicopathological parameters and survival

Factors (N)		I-LVD		P-LVD	
		mean ± SD	*P*	mean ± SD	*P*
Age	<60 (33)	12.60 ± 5.32	N.S.	10.52 ± 3.22	N.S.
	>60 (33)	11.99 ± 3.15		11.53 ± 3.99	
Sex	Male (47)	12.31 ± 4.79	N.S.	11.06 ± 3.91	N.S.
	Female (19)	12.24 ± 3.11		10.88 ± 2.83	
Extent of resection	Total gastrectomy (16)	13.78 ± 5.62	N.S.	12.36 ± 5.30	N.S.
	Subtotal gastrectomy (50)	11.82 ± 3.81		10.59 ± 2.87	
Tumor location	Upper 1/3 (12)	12.01 ± 3.30	N.S.	11.74 ± 5.40	N.S.
	Middle 1/3 (21)	13.49 ± 5.18		11.26 ± 3.63	
	Lower 1/3 (32)	12.33 ± 4.37		10.63 ± 2.92	
Tumor size	<5cm (36)	12.20 ± 4.98	N.S.	10.55 ± 3.61	N.S.
	>5cm (30)	12.40 ± 3.53		11.56 ± 3.62	
Lauren classification	Intestinal type (31)	11.20 ± 3.06	0.048*	10.10 ± 2.74	N.S.
	Diffuse type (35)	13.26 ± 5.09		11.55 ± 4.23	
Differentiation	Differentiated (31)	11.03 ± 2.80	0.021*	10.49 ± 2.74	N.S.
	Undifferentiated (35)	13.41 ± 5.15		11.47 ± 4.25	
Tumor stage (AJCC 7^th^ ed.)	T1 (18)	10.22 ± 3.53	0.024*	9.87 ± 3.55	N.S.
	T2 (11))	12.95 ± 4.09		11.70 ± 3.64	
	T3 (26)	12.23 ± 3.95		11.05 ± 3.74	
	T4 (11)	15.16 ± 5.37		12.18 ± 3.37	
LVI	Negative (37)	10.96 ± 3.80	0.004*	10.18 ± 3.71	0.028*
	Positive (29)	13.99 ± 4.48		12.12 ± 3.28	
Node stage (AJCC 7^th^ ed.)	N0 (36)	11.26 ± 3.84	0.052	10.00 ± 2.77	0.040*
	N1 (3)	3.34 ± 2.20		10.25 ± 0.55	
	N2 (7)	13.51 ± 3.79		12.38 ± 1.84	
	N3 (20)	14.16 ± 5.00		12.67 ± 4.38	
Tumor node metastasis stage (AJCC 7^th^ ed.)	I (20)	10.45 ± 3.93	0.029*	9.81 ± 3.02	0.061
	II (30)	12.27 ± 3.58		10.88 ± 3.18	
	III (18)	14.17 ± 5.14		12.56 ± 4.48	
DFS (month)	LVD-Low (38)	57.82 ± 3.07	N.S.	59.31 ± 2.77	0.037*
	LVD-High (28)	51.45 ± 4.84		51.21 ± 4.81	
OS (month)	LVD-Low (38)	61.70 ± 2.62	0.031*	60.06 ± 2.70	0.088
	LVD-High (28)	52.60 ± 4.44		55.96 ± 4.14	

*LVI*, lympho-vascular invasion; *DFS*, disease free survival; *OS*, overall survival

*, *P* <0.05; N.S., not significant; data are expressed as means ± SD

### Immunohistochemistry and assessment of LVD

Tumor specimens were prospectively collected at the tissue bank of the hospital after operation. Immunohistochemical staining was performed on 4-μm thick samples that had been fixed in 10 % formalin and embedded in paraffin. The paraffin was solubilized and removed with xylene and the sections were rehydrated. Endogenous peroxidase was blocked with 3 % hydrogen peroxide for 10 min. Immunoperoxidase staining was performed using a streptavidin-biotin peroxidase method. Antigen retrieval was performed using 0.01 M sodium citrate buffer through microwave processing for D2-40 antibody, an immunostaining marker for lymphatic endothelium. Non-specific antibody binding was blocked with normal rabbit serum. The slides were incubated with primary antibodies for D2-40 (1:100 diluted; DAKO Cytomation, Glostrup, Denmark). Antigen-antibody reaction was visualized using 3-amino-9-ethylcarbazole substrate (AEC, NeoMarkers, Fremont, CA, USA) as chromogen. The slides were counterstained with Mayer’s hematoxylin and mounted. The immune-staining of all 66 selected formalin fixed paraffin embedded tissues were performed in a way under the same conditions.

I-LVD was measured at the tumor center while P-LVD was measured in the periphery within 2 mm of tumors adjacent to the invasion front. The two values were assessed separately. LVD was detected by immunostaining with D2-40. First, we selected five hot spot areas with highly D2-40 positive vessels in peritumoral sections and intratumoral sections were identified by scanning the sections at 40 X magnification. Next, the number of D2-40 positive vessels was counted in randomly selected three fields in each hot spot area at 200 X magnification [24]. The mean value for the three fields was taken as the LVD. The 66 cases were divided into two groups according to the mean LVD level, either I-LVD or P-LVD group. Scoring and counting were performed independently by two pathologists without knowledge of clinicopathological data or survival of patients.

### Statistical analysis

Analyses were performed using the SPSS statistical software program for Windows version 21 (SPSS Inc., Chicago, IL, USA). Comparison of the means was performed using Student’s *t*-test and one-way ANOVA, followed by Tukey’s multiple comparison test for continuous variables. The survival data for both groups were analyzed by means of the Kaplan-Meier method and the log-rank test was used for the assessment of the difference between the two groups. Two-sided *P* <0.05 was considered a statistically significant difference.

## Results

### Intratumoral and peritumoral lymphatic characteristics in gastric cancer

**Fig. 1:**
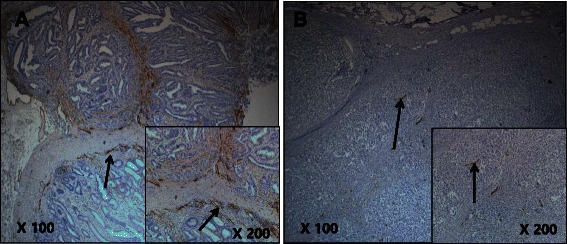
Intratumoral lympho-vascular density (I-LVD). **a** intestinal type, **b** diffuse type. Arrows indicate lymphatics

**Fig. 2:**
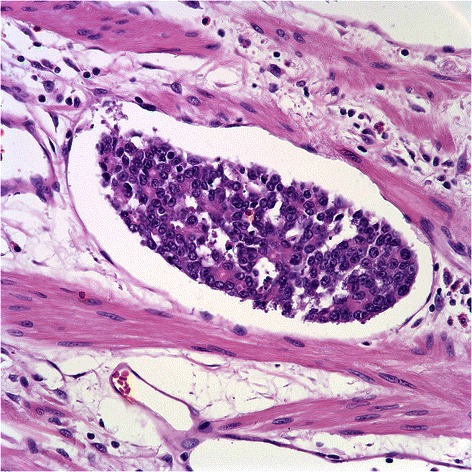
Intratumoral lymphatics containing tumor (Lymphovascular invasion)

**Fig. 3:**
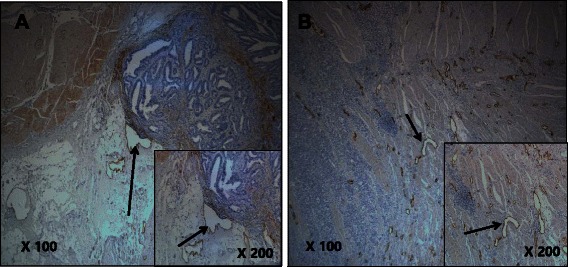
Peritumoral lympho-vascular density (P-LVD). **a** intestinal type, **b** diffuse type. Arrows indicate lymphatics

The D2-40-positive lymphatic vessels had irregular morphology and thin-walled lumens. Lymphatic vessels in gastric tissues were mostly located in the layer of submucosa. Intratumoral lymphatic vessels were usually collapsed, small, and irregular in all intestinal and diffuse types (Fig. 1a and b), but some non-collapsed lymphatics had open lumens and occasionally contained invading tumor-cell clusters (Fig. 2). The peritumoral lymphatic vessels were enlarged with dilated lymphatic cavities, regardless of their Lauren classification (Fig. 3a and b). Overall, the mean I-LVD was higher than the mean P-LVD (12.29 ± 4.35 vs. 11.01 ± 3.62 [±SD throughout], *P* = 0.025). In addition, the mean I-LVD of the intestinal and diffuse subtypes was significantly different (11.20 ± 3.06 vs. 13.27 ± 5.09, respectively, *P* = 0.048). However, the P-LVD of the two subtypes was not significantly different..

### Correlations of I-LVD and P-LVD with clinicopathological parameters and prognosis

**Fig. 4:**
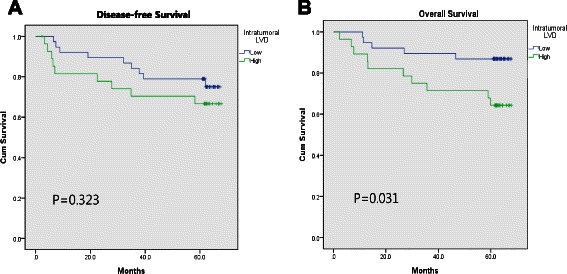
Survival curve according to the intratumoral lympho-vascular density (I-LVD). **a** Disease-free survival, **b** overall survival

**Fig. 5:**
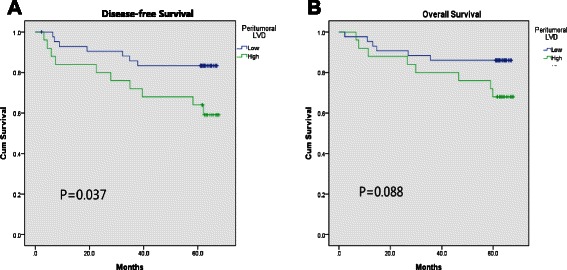
Survival curve according to the peritumoral lympho-vascular density (P-LVD). **a** Disease-free survival, **b** overall survival

The correlations of I-LVD and P-LVD with clinicopathological parameters are shown in Table 1. Neither I-LVD nor P-LVD correlated with age, sex, extent of resection, tumor location, or tumor size. In addition to the association of I-LVD with the diffuse type of cancer, it was also significantly associated with the undifferentiated type (*P* = 0.021), tumor stage (T-stage; *P* = 0.024), LVI (*P* = 0.004), tumor node metastasis (TNM) stage (*P* = 0.029), and poor overall survival (*P* = 0.031; refer also to Fig. 4 a and b). Although it failed to reach statistical significance, the data showed a trend for I-LVD to associate with node (N)-stage (*P* = 0.052). In comparison, P-LVD had a significant correlation with LVI (*P* = 0.028), N-stage (*P* = 0.040), poor disease-free survival (*P* = 0.037; also refer to Fig. 5a), and a tendency for association with TNM-stage (P = 0.061) and overall survival (*P* = 0.088, Fig. 5b). Lauren classification, differentiation, and T-stage were not correlated with P-LVD.

We suggest that these results are consistent with different biological roles for I-LVD and P-LVD in gastric cancer. I-LVD was more closely correlated with depth of invasion than it was with lymph node metastasis, while P-LVD had a strong relationship with lymph node metastasis rather than depth of invasion. Both parameters correlated with TNM stage and oncological long-term survival. Thus, both intratumoral lymphatics and peritumoral lymphatics may contribute to gastric cancer progression and prognosis but in different ways.

## Discussion

A study by Padera and coworkers, with an in vivo animal model, has conclusively established that peritumoral lymphatics are predominantly responsible for the uptake ability of cancer cells during metastasis [16]. However, the role of intratumoral versus peritumoral lymphatics in the pathology of primary human tumors [3, 5, 8, 12–15] has not been so convincingly demonstrated. To date, there have been seven studies comparing the values of I-LVD and P-LVD in lymph node metastasis. In three of them, P-LVD was more highly correlated with lymph node metastasis and a poor prognosis than was I-LVD [17–19], although other studies do not confirm the correlation. Lee et al. [25] reported that I-LVD in early gastric cancer was related to lymph node metastasis, while P-LVD was not, in either early or late stages of the disease. Gao et al. [26] found that both I-LVD and P-LVD were associated with lymph node metastasis in early gastric cancer, but only P-LVD was correlated with it in advanced stages. Raica et al. [21] reported that both I- and P-LVD were related to lymph node metastasis and a poor prognosis, but in yet another study, there was no correlation between LVD in either location and lymph node metastasis or prognosis [22]. Based on our results, we suggest that P-LVD is more important in lymph node metastasis than is I-LVD. Some association of I-LVD with lymph node metastasis was indicated and although it failed to reach statistical significance (*P* = 0.052), the data do not exclude the possibility that I-LVD influences lymph node metastasis.

The distribution of lymphatic channels in tumors also appears to vary by the organ affected. Few lymphatic channels in breast cancers have been identified in intratumoral areas, with the majority located around the tumor periphery [27]. However, increased LVD within the tumor and in peritumoral areas has been observed in cutaneous melanoma [28]. In our study, I-LVD was generally higher than P-LVD, in agreement with Lee et al. [20]. In addition, I-LVD was positively correlated with the depth of tumor invasion. These findings indicate that intratumoral lymphatic channels are the product of neovascularization by tumor cells rather than simple entrapment of pre-existing, normal lymphatic channels. LVD in both locations was correlated with TNM stage and led to a poor prognosis. This may indicate that high LVD in either location contributes to gastric cancer progression, but that they act in different manners.

The results indicate a correlation between P-LVD and TNM stage although they did not quite reach significance (*P* = 0.061). Likewise, we observed some clear trends in our analyses of long-term survival, but the statistical analysis requires that they should be interpreted cautiously. Nonetheless, the results are intriguing and we believe the failure to detect clear differences is due to the small sample size and that both I-LVD and P-LVD are important in cancer progression and the poor prognosis of gastric cancer patients.

Angiogenesis inhibitor (Avastin®, bevacizumab) has been used as a molecular targeting agent in the treatment of colon cancer and renal cell carcinoma [29, 30]. Although it was ineffective in the treatment of gastric cancer in the AVAGAST trial, the results cannot be considered conclusive because the study was analyzed without the biomarker’s classification [31]. In addition, the REGARD trial showed that the VEGFR-2 inhibitor prolonged the survival of patients with advanced gastric cancer [32]. Similar to the growing awareness of the importance of angiogenesis, awareness of the importance of lymphangiogenesis is emerging. In particular, in the case of tumors in which prognosis is dependent on lymph node status and in which lymph node metastasis is a major biological process leading to distant tumor propagation, lymphangiogenesis inhibition is of utmost clinical importance. Thus, continued research on lymphangiogenesis is critical.

We have some limitations to draw a solid conclusion in this study. One of them is a selection bias. We selected 66 cases arbitrarily from 365 patients for IHC staining, although we matched TNM stages according to Lauren’s classification to remove the effect of tumor aggressiveness. Small samples must be one of limitations too. However, our results could suggest some conceptual important points. In this study, we used D2-40 for staining of lymphatics. D2-40 is a commercially available monoclonal antibody directed against human podoplanin a transmembrane mucoprotein that is expressed in lymphatic endothelial cells. Many studies indicate that D2-40 is specific for lymphatic invasion and LVD in human cancers, including gastric cancer [33–37]. However, some studies reported that D2-40 could be expressed in other tissues such as seminoma, epithelioid, adrenal cortical tumor, Kaposi sarcoma, adnexal tumors of the skin [38–43]. D2-40 immunoreactivity is restricted to lymphatic endothelium, not blood vessels. Therefore, the conjunction with specific vascular marker, such as CD31 or CD34, which highlights both blood vessel and lymphatic endothelium was suggested to detect lymphatics specifically [44]. Wang et al. performed immune-staining of lymphatics with D2-40 and CD31 in gastric cancer tissue and showed that D2-40 showed exclusively stained lymphatic endothelium [18]. Consistently, our study also demonstrated that D2-40 was a good lymphatic endothelial maker in gastric cancer tissue.

In conclusion, although we should be cautious due to the small sample size, P-LVD might have a more important role in lymph node metastasis than I-LVD, while I-LVD was associated with depth of tumor invasion. Collectively, high LVD in both locations contributed to gastric cancer progression and prognosis; thus, lymphangiogenesis inhibition should be considered an important target of therapy for treatment of gastric cancer.

## Conclusions

We conclude that P-LVD was significantly associated with lymph node metastasis, while I-LVD was more associated with depth of tumor invasion. However, both LVDs contributed to gastric cancer progression and prognosis.

## Abbreviations

I-LVD: Intratumoral lymphatic vessel density
P-LVD: Peritumoral lymphatic vessel density
LVD: Lymphatic vessel density
LVI: Lymphatic vascular invasion
